# Emerging Role of Immune Checkpoint Blockade in Pancreatic Cancer

**DOI:** 10.3390/ijms19113505

**Published:** 2018-11-07

**Authors:** Shravanti Macherla, Shachar Laks, Abdul Rafeh Naqash, Anushi Bulumulle, Emmanuel Zervos, Mahvish Muzaffar

**Affiliations:** 1 Department of Medicine, Division of Hematology/Oncology, East Carolina University Brody School of Medicine, Greenville, NC 27834, USA; MACHERLAS17@ECU.EDU (S.M.); NAQASHA16@ECU.EDU (A.R.N.); BULUMULLEA17@ECU.EDU (A.B.); 2 Department of Surgery, Division of Surgical Oncology, East Carolina University Brody School of Medicine, Greenville, NC 27834, USA; LAKSS17@ECU.EDU (S.L.); zervose@ecu.edu (E.Z.)

**Keywords:** pancreatic adenocarcinoma, programmed death ligand, microsatellite instability

## Abstract

Immune checkpoint blockade (ICB) with programmed cell death protein-1(PD-1)/programmed death ligand -1(PD-L1) antibodies has revolutionized the management of several cancers, especially non-small cell lung cancer, melanoma, urothelial, and renal cancer. Pancreatic ductal adenocarcinoma (PDAC) is one of the most aggressive cancers associated with high morbidity and mortality. Based on available data, it’s obvious that ICB has limited success in PDACs, which can be explained by the low immunogenicity and immunosuppressive tumor microenvironment of these tumors. In this review article, we focus on PD-L1 expression and microsatellite instability (MSI) in PDAC, and their roles as prognostic and predictive markers. We also discuss data supporting combination therapies to augment cancer immunity cycle. Combining anti-PD-1/PD-L1 agents with other modalities such as vaccines, chemotherapy, and radiation could potentially overcome resistance patterns and increase immune responsiveness in PDAC.

## 1. Introduction

Pancreatic ductal adenocarcinoma (PDAC) is a highly lethal malignancy with a dismal five year overall survival(OS) rate of 8.5%, making it the third leading cause of cancer-related death, with an estimated 55,400 new cases and 44,330 deaths in the United States in 2018 [1]. Even loco-regional PDAC has a poor prognosis, with a 5-year OS of 10–35% while metastatic disease, which accounts for more than 50%, has a grim 3% 5-year OS rate [2]. PDAC poses several treatment challenges, including late presentation and a unique tumor microenvironment characterized by dense desmoplasia and intense infiltrations of immunosuppressive cells [3,4] contributing to chemotherapy resistance. Surgery is the only curative option, but only 10–15% of patients have resectable disease at the time of diagnosis. For most patients with advanced PDAC, systemic chemotherapy has been the mainstay of treatment for disease control. Over last several decades, various chemotherapy regimens have shown minimal incremental benefit.

Despite the modest improvements with combination chemotherapy regimens, prognosis for pancreatic cancer remains very poor, and there is an unmet need to identify new targeted therapies [5,6,7,8]. So far, studies exploring therapies targeting KRAS, BRAF, BRCA 1 and 2, ATM, CDKN2A mutations have not shown any meaningful survival benefit in PDAC. Conversely, PD-1/PD-L1 inhibitors have shown promising results in various solid malignancies including other gastrointestinal malignancies, making their incorporation into the treatment paradigm of an aggressive malignancy like PDAC very appealing. In this review article, we will discuss the available evidence of programmed cell death protein-1(PD-1)/programmed death ligand-1(PD-L1) blockade in pancreatic cancer and the potential roles of PD-L1 expression and microsatellite instability (MSI) as prognostic/predictive biomarkers.

## 2. The PD-1/PD-L1 Pathway

The immune system has three primary roles in the prevention of tumors, of which perhaps the most important may be immune surveillance. In this function, the immune system can specifically identify and eliminate tumor cells by their expression of tumor-specific antigens or molecules [9]. Currently, the updated concept of tumor immunoediting is more relevant, as it explains tumor development that occurs despite a functioning immune system. This concept is further divided into 3 phases: elimination, equilibrium, and escape [10]. By interaction with a host immune system, the malignant cells may determine its fitness for survival and growth, thereby aiding the malignant cell to evade the tumor suppressor function of the immune system, that is, if the immune response fails to eliminate the tumor.

Immune checkpoint regulators, such as programmed cell death-1 and cytotoxic T lymphocyte antigen-4 (CTLA-4), belong to a class of co-inhibitory receptors present mainly on T cells. Under physiological homeostasis, binding of these checkpoints to their cognate ligands regulates the balance between self-tolerance and immunopathology [11]. Direct and indirect utilization of these co-inhibitory pathways by tumors results in their ability to evade an immune attack. This mechanism, termed adaptive immune resistance, facilitates tumor growth and propagation. The PD-1 pathway is an essential inhibitory mechanism regulating T cell exhaustion. PD-1 and one of its ligands, PD-L1 (also known as B7-H1), constitute a major tolerance mechanism in tumors. Immune-checkpoint antibodies directed against PD-1 and PD-L1 restore antitumoral immunity by augmentation of an endogenous immune response [12].

## 3. PD-L1 Expression and Prognostic Significance in Pancreatic Cancer

PD-L1 expression and its clinical significance have been studied extensively in solid tumors. PD-L1 is expressed on the surface of tumor cells in various malignancies, including carcinoma of head and neck, melanoma, lung, esophagus, thyroid, thymus, breast, gastrointestinal, colorectal, liver, pancreas, kidney, bladder, ovary, and skin [13,14,15,16,17,18,19]. PD-L1 is rarely expressed on normal tissue but exclusively expressed in tumor cells, indicating that this selective expression of PD-L1 may have some association with outcomes in various cancers [20]. Some cancers, including hepatocellular carcinoma, pancreatic cancer, gastric cancer, renal cell carcinoma, and esophageal cancer, have an immunosuppressive tumor microenvironment with a high PD-L1 expression, which in turn inhibits cytotoxic effects of activated T-cells [21]. This may explain why overexpression of PD-L1 in these tumors is generally associated with poorer prognosis. Interestingly, in lung cancer, melanoma, and colorectal carcinoma, PD-L1 expression has both positive and negative predictive values [22]. Considering that PD-L1 overexpression is associated with variable prognosis, this raises the speculation of other mechanisms, in addition to variations in PD-L1 expression within the tumor microenvironment, as some of the critical determinants of outcomes in certain tumors.

Several studies have evaluated the expression of PD-L1 in PDAC. In a meta-analysis by Gao et al. [23], which analyzed nine studies, the PD-L1 positive rate in PDAC ranged from 19% to 62.5%. The PD-L1 positive rate measured by immunohistochemistry (IHC) was higher than by PCR. As shown by some, IHC-based detection of PD-L1 has limitations [24]. First, IHC-based detection has technical challenges and may not yield accurate results for PD-L1 expression. Secondly, detection of PD-L1 expression is affected by temporal and spatial factors [25]. Hence, single point evaluation may not reflect the actual condition and may require multiple site sampling to determine true PD-L1 expression.

Six studies (Table 1) focusing on PD-L1 expression and prognosis in PDAC demonstrated that PD-L1 expression is associated with poor prognosis. Nomi et al. [15] examined 51 patients with PDAC, using IHC with a 10% cut-off value of immunoreactivity per high power field to define the PD-L1 expression. PD-L1 positive patients (*n* = 20) had a significantly poorer prognosis than PD-L1 negative patients (*p* = 0.016). One-year post-operative survival rate was 33.5% and 60.3% in PD-L1 positive and PD-L1 negative patients, respectively. Wang et al. [26] examined 81 patients with PDAC using IHC, with a 5% cut-off value to define the PD-L1 expression. They observed that PD-L1 inhibits activation of T lymphocytes, which promotes tumor evasion and T cell exhaustion. This study concluded that PD-L1 expression could act as an independent prognostic factor after adjusting for pathological and TNM stages. Chen et al. [27] examined 63 cases of pancreatic cancer tissue for PD-L1 (>50% of patients expressed PD-L1) and other inhibitory costimulatory molecules using IHC asdefined by >10% clear staining among 1000 tumor cells/section. The expression of PD-L1 and other B7 family molecules resulted in tumor growth and decreased survival. Loos et al. [28] investigated the expression pattern using reverse transcription PCR (RT-PCR) in 40 human pancreatic cancer tissue samples, and the clinical significance of B7 family molecules including PD-L1 in PDAC. Among the investigated molecules, only PD-L1 showed prognostic relevance. Postoperatively, the median survival in patients with low PD-L1 expression was 24 months, in comparison to 10 months for high PD-L1 expression (*p* < 0.0001). Geng et al. [29] examined 40 pancreatic cancer specimens for PD-L1 and IL-10 expression using RT-PCR. Analysis of the relationship between PD-L1 and tumor clinicopathological characteristics showed that positive PD-L1 expression was associated with reduced tumor differentiation and advanced tumor stage. Birnbaum et al. [30] conducted a retrospective study, wherein they analyzed PD-L1 mRNA expression in 453 pancreatic cancer samples. Nineteen percent of the cancer samples had upregulation of PD-L1 expression. PD-L1 positive pancreatic cancer samples displayed evidence of lymphocyte exhaustion and was associated with shorter disease-free survival and overall survival in multivariate analysis. Hence, PD-L1 overexpression can serve as a novel biomarker for prognostication and a potential target for the treatment of PDAC with PD-1/PD-L1 inhibitors.

## 4. Mismatch Repair (MMR) Deficiency and Hypermutation in Pancreatic Cancer

The mismatch repair (MMR) system plays a pivotal role in the repair of DNA sequence mismatches during replication. Defects in the MMR system (dMMR) or loss of function of one of the MMR proteins (MLH1, MSH2, MSH6 and PMS2) causes errors in DNA replication, leading to the high burden of mutations that accumulate in microsatellites (short tandem repeats that are prone to DNA replication errors), resulting in MSI [31]. A defective MMR system leads to an accumulation of somatic mutations, resulting in a higher neoantigen load, which promotes proinflammatory cytokines and activation of T cells. Increased neoantigens and cytotoxic T cell recruitment contributes to the immunogenicity of dMMR tumors and hence, sensitivity to immunotherapy [32].

Tumors with dMMR/MSI can develop either as a result of a germline mutation in the MMR gene, i.e., Lynch syndrome, or more commonly, as epigenetic inactivation of the MLH1 gene [33]. Tumors are classified as MSI-high (MSI-H) if they have two or more of the five microsatellites markers to show instability, MSI-low (MSI-L) if only one of the markers shows instability, and MSIstable (MSS) if none of the markers show instability [34]. The clinical implications of MSI-L are unclear. Tumor mutational load (TML) is defined as the total number of mutations per coding area of a tumor gene, and tumors that are MSI-high (MSI-H) typically have high TML [35]. Evidence suggests that tumors with high TML status have increased sensitivity to immunotherapy [36].

Due to the recently approved site agnostic indication for pembrolizumab, a recent update in the National Comprehensive Cancer Network guidelines encourage MSI testing for locally advanced and metastatic PDAC [37]. The DNA mismatch repair or microsatellite instability in PDAC can be assessed by IHC and PCR, respectively. MSI testing can be performed on fresh or frozen samples using PCR techniques which primarily detect instability [33]. Depending on the PCR test results, tumors are classified as MSI-H, MSI-L, or MSS as described above. MMR protein expression can also be detected by IHC, as a loss of expression in ≥1 mismatch repair proteins this is labelled dMMR. The advantages include wide availability, cost effectiveness, and rapid detection of loss of specific mismatch gene product, and thereby can direct germline testing to that gene. In most instances, MMR testing by IHC and MSI testing by PCR are concordant [38]. Another test that is clinically available and has therapeutic relevance is next generation sequencing (NGS), which can help measure tumor mutational burden, MSI status, specific actionable mutations, and germline mutations within the tumor.

The prevalence of dMMR/MSI in PDAC is likely very low, but literature in this regard is inconsistent. Hu et al. evaluated MMR status on 833 PDAC cases using NGS assays targeted to perform deep sequencing of all exons and selected introns. They found dMMR in PDAC is a rare event occurring at a frequency of 0.8% (7/833) and all 7 patients with dMMR PDAC were found to have Lynch syndrome [39]. Kim and colleagues conducted a prospective analysis of PD-L1 and MMR IHC on 430 patients (6/430 with PDAC) with advanced gastrointestinal and genitourinary cancers. Among the 394 evaluable for MMR/MSI status, 18 patients had dMMR tumors. The dMMR was most common in gastric cancer and colorectal (11.1%), and nearly 0% in PDAC [40]. However, some others have reported MSI/dMMR in PDAC to be as high as 22% [41]. The discordance with regard to its prevalence in pancreatic cancers could be confounded by variabilities in histology, sample sizes, and more so due to non-standardized testing and evolving detection methods.

## 5. MSI as a Prognostic Marker and Correlation of MSI, PD-L1 and Tumor Mutational Load (TML) in Pancreatic Cancer

The favorable prognosis of MSI positive tumors compared to MSI negative tumors such as colorectal cancer [42], gastric cancer [43], and cancer of papilla of Vater [44] is well established. Interestingly, smaller studies suggest that the prognosis of MSI positive breast cancer [45] and non-small cell lung cancer [46] is worse than MSI negative tumors. Though limited literature exists, PDAC patients with dMMR have been reported to have prolonged survival rates. Nataka et al. [47] examined the prognostic impact of microsatellite instability in 46 subjects that underwent resection of pancreatic cancer. DNA was analyzed using PCR techniques and revealed eight patients (17.4%) with MSI positivity. Univariate analysis showed MSI positives patients had significantly longer survival time compared to MSI negative patients (median survival term, 62 months versus 10 months, respectively; *p* = 0.011). Yamamoto et al. [48] analyzed 100 sporadic and 3 hereditary PDACs for MSI. Of the 100 sporadic cases, 13 were MSI-H (13%), 13 were MSI-L (13%), and 74% were MSS. All the three hereditary tumors (Lynch syndrome) were MSI-H. Patients with MSI-H tumors had significantly prolonged survival times compared to patients with MSI-L and MSS tumors (*p* value of 0.0057). The etiology for improved survival in MSI-H resected PDAC tumors is unclear, but thought to be due to enhanced immunogenicity in these tumors that are deficient in DNA replication error repair.

Increased expression of PD-L1 and dMMR/MSI status on tumors may be useful predictive response biomarkers for immunotherapy. The overlap of PD-L1 and dMMR has not been extensively studied. Kim and colleagues [40] further analyzed 365 patients for both PD-L1 and MLH1/MSH2 expression. PD-L1 expression was seen in 38.9% (7/18) of dMMR tumors and 15.2% (15/376) of MMR proficient tumors, hence implying a significant association between PD L1 expression and MLH1/MSH2 loss (*p* = 0.01). Theoretically, dMMR tumors tend to have a high mutational burden with increased neoantigen expression and tumor infiltrating lymphocytes (TILS), all of which are expected to increase PD-L1 expression [49]. The correlation between TML, dMMR, and PD-L1 was studied by Salem et al. [35], who analyzed 4125 tumors from 14 different gastrointestinal cancers, including 870 patients with PDAC. They found a lower prevalence (1.4%) of TML-high (defined as greater than or equal to 17 mutations/MB) in PDAC. PD-L1 expression was positive in 11.1% (1/9) of MSI-H PDACs and 8.6% (69/806) of MSS PDACs. In the pancreatic adenocarcinoma subgroup, among patients who had either MSI-H or MSI-L, the majority had TML-low, except for a very small percentage that had both MSI-H and TML-high. In MSS PDACs, only 1.4% were TML-high. Future clinical trials would be required to study whether the integration of PD-L1, MSI, and TML could serve as a better predictive marker of response to immunotherapy in PDAC.

## 6. PD-1/PD-L1 Inhibitors in the Treatment of Pancreatic Cancer

Currently, the US Food and Drug Administration (FDA) has approved PD-1 inhibitor pembrolizumab for the treatment of unresectable or metastatic solid tumors characterized by MSI-H status or dMMR that has progressed following prior treatment. The pivotal data that drove the FDA approval for the site agnostic indication of Pembrolizumab in treatment of MSI-H tumors [50] is based on the results from five single-arm clinical trials that enrolled a total of 149 patients, including Keynote (KN)-016 (*n* = 58), KN-164 (*n* = 61), KN-012 (*n* = 6), KN-028 (*n* = 5) and KN-158 (*n* = 19). Patients with colorectal cancers constituted a significant proportion in the combined cohort of these five studies. Only a total of six patients had pancreatic carcinoma, with a significant majority having a response to ICB with anti-PD-1 therapy. The pivotal phase 2 study conducted by Le et al., [32] that evaluated 86 patients with 12 different dMMR cancers, including pancreatic cancer, demonstrated the efficacy of PD-1 inhibition with objective radiographic responses and complete responses in 53% and 21% of patients, respectively. Keynote (KN)-164 [51] and KN-158 (NCT02628067) assessed the efficacy of pembrolizumab in patients with MSI-H tumors who had progressed on ≥2 prior lines of therapy and showed robust activity against MSI-H tumors in heavily pretreated individuals. KN-164 enrolled 61 patients with MSI-H colorectal cancer (CRC). KN-158 enrolled 19 patients and included 2 patients with pancreatic cancer. Median follow up was 4.5 months for MSI-H non-CRC, and overall response rate for MSI-H non-CRC was 42.9%, with 8 confirmed responses (per RECIST criteria) and 1 unconfirmed response.

Interestingly, both studies showed the positive benefit of ICB in patients with MSI-H tumors irrespective of PD-L1 status. Another PD-1 inhibitor, nivolumab, has been approved for MSI-H/dMMR metastatic colorectal cancer that has progressed following first-line treatment. It was also approved for treatment of hepatocellular carcinoma in patients who have been previously treated with sorafenib [52]. These data strongly suggest that compared to PD-L1, dMMR is as a robust determinant of response to ICB, with no significant discrepancies seen among studies.

With the advent of ICB, there has been a paradigm shift in the treatment of various other malignancies, particularly non-small cell lung cancer and melanoma. Unfortunately, the success of immunotherapy seen in certain tumor subtypes hasn’t been replicated in the treatment of PDAC, except in patients with dMMR/MSI-H tumors (Table 2). In early clinical trials, single-agent anti-PD-1/PD-L1 or anti-CTLA-4(Cytotoxic T lymphocyte antigen-4) agents have been ineffective in the treatment of PDAC.

Some of the plausible reasons contributing to resistance to immunotherapy can be explained by the low immunogenicity of PDACs, attributed to low mutational burden [53] and fewer neoantigens, therefore, creating an environment which is less easily recognized by the immune system. In the study from Alexandrov et al. [54], the mutational burden of 7042 cancers were analyzed. It was found through genomic analysis that melanomas had an average of 511 coding mutations, but PDACs only had an average of 61 coding mutations. The other feature of PDACs that makes them less responsive to immune therapy is the hypothesis of an immunosuppressive tumor microenvironment characterized by dense desmoplasia, infiltration by immune suppressive myeloid cells and very few T cells [55]. Furthermore, PDAC cells can evade immune attack by downregulating the Fas receptor and induce apoptosis of cytotoxic T cells by intensifying the Fas ligand expression [56].

## 7. Future Directions

Pancreatic cancer continues to be one of the most aggressive cancers and is traditionally considered to be a nonimmunogenic cancer. A possible explanation is a limited effector T cell in the tumor, along with increased regulatory T cells, in a very immunosuppressive microenvironment. The promise of potentiating the immunogenicity of pancreatic cancer is based on initial results from combination therapies i.e., conventional cytotoxic agents, radiation therapy, and therapeutic vaccine with immune checkpoint inhibitors, with the premise that the addition of these novel combinations to ICB can potentially augment the anti-tumor immunity in an immunologically inert tumor microenvironment that is characteristic of PDACs. This concept revolves around the fact that the addition of these agents to ICB likely facilitates the conversion of an immune desert to an immune-active microenvironment. Therapies to augment the activity of immunotherapy and that overcome the immunosuppressive environment of PDACs can be broadly classified into three categories.

Firstly, agents that enhance naïve T cell activation include chemotherapy (gemcitabine), radiation, vaccination (GVAX, CRS-207), and agonist CD-40 agents. Animal models have provided evidence that gemcitabine can potentially augment antitumor immunity [57]. Plate and colleagues evaluated the effects of gemcitabine on T cell subsets, B cells, myeloid dendritic precursor cells, and memory and naïve T cells in 10 patients with PDAC [58]. Their study confirmed that gemcitabine might suppress memory T cells, but augments naïve T cell function, hence they concluded that gemcitabine was not immunosuppressive, and it may enhance responses to immunotherapy and certain vaccines. Early preclinical studies have shown antibodies against PD-1 and PD-L1 induced a significant antitumor effect in a mouse pancreatic tumor model, which was further enhanced when administered in combination with gemcitabine [15]. Currently, studies to evaluate the potential value of combining anti-PD-L1 antibody and gemcitabine are ongoing. The other option is combining PD-1/PD-L1 inhibitors with a pancreatic cancer vaccine (GVAX). The vaccine is composed of irradiated, allogenic pancreatic tumor cells that express granulocyte-macrophage colony stimulating factor (GM-CSF). Also, when GVAX was used with low dose cyclophosphamide in 39 patients with PDAC, it resulted in the formation of intratumoral lymphoid aggregates and T cell infiltration in 33 of 39 patients, thus providing an example of a vaccine based immune therapy converting a non-immunogenic tumor into an immunogenic tumor [59]. But a phase 2b, randomized, multicenter study (ECLIPSE) comparing GVAX pancreas and CRS-207 to chemotherapy did not show any survival benefit in the combination arm (cyclophosphamide plus GVAX plus CRS-207) when compared to chemotherapy (physician’s choice of single-agent chemotherapy) [60]. These results are in contrast to the earlier phase 2a study and ongoing studies are combining CRS-207 with immunotherapy to enhance the immune responsiveness of PDAC. A similar proof of concept, albeit having much better success of converting an immune evasive microenvironment to an immune permissive one, has been used in metastatic melanoma where combinations of talimogene laherparepvec (T-VEC), an oncolytic virus derived from an attenuated recombinant type-1 herpes virus with pembrolizumab [61] and ipilimumab [62], has shown promising outcomes in early phase clinical trials. This seems to suggest that unlike melanoma, certain intrinsic properties of the PDAC tumor cells that create a significant degree of immune inertness [63] may prevent generation of a robust anti-tumor response. Currently, numerous randomized clinical studies are evaluating the combinations of anti-PD-1 therapy and GVAX vaccine in PDAC. Radiotherapy and CD40 agonists have also demonstrated the ability to augment various aspects of T cell immunity and are currently being studied in combination with PD-1/PD-L1 inhibitors. (Table 3) Secondly, agents targeting the immunosuppressive microenvironment including radiotherapy, Janus kinase (JAK) inhibitors and phosphoinositide-3-kinase (PI3K) inhibitors [64] that have shown promise in preclinical studies are being further evaluated. Lastly, agents to break down the desmoplastic stroma, such as PEGPH20, were assessed in a phase 2 study (HALO 202) comparing PEHPH20 plus nab-paclitaxel/gemcitabine versus nab-paclitaxel/gemcitabine in patients with untreated, metastatic PDAC. Results from this study showed improvement in progression free survival PFS (*p* = 0.049) in the PEGPH20 plus chemotherapy arm, especially in those with high hyaluronan accumulation [65]. Ongoing studies are combining anti-PD-1/PD-L1 agent with vaccines, radiation, and chemotherapy in the treatment of PDAC, some of which are listed below (Table 3). The ongoing trials are focusing on augmenting various steps of the cancer immunity cycle by the combination therapies.

## 8. Conclusions

Despite recent advances in combination chemotherapy regimens, PDAC remains a deadly malignancy with dismal outcomes. ICB with PD-1 and PD-L1 antibodies have demonstrated durable response rates, predominantly in immunogenic tumors, and unfortunately, pancreatic cancer does not belong to that category. PD-L1 expression and MSI status is prognostic and predictive of immune responsiveness in many malignancies. Single agent immunotherapies are ineffective in pancreatic cancer. Preliminary results are more promising for a combined integrated approach utilizing, chemotherapy, radiation therapy, immunotherapy, and other targeted agents to augment the immune response in PDAC. This strategy may provide some hope, which has been elusive in pancreatic cancer.

## Figures and Tables

**Table 1:** Studies reporting programmed cell death protein-1 (PD-L1) expression rates and outcomes in patients with pancreatic ductal adenocarcinoma (PDAC).

Study	Number of Patients with PDAC	PD-L1 Detection Method	PD-L1 Positive Rate	OS/DSS HR (95% CI)
Nomi et al. [15]	51	Protein (IHC)	39.2%	2.66 (1.21–5.85)
Wang et al. [26]	81	Protein (IHC)	49.4%	2.08 (1.17–3.72)
Chen et al. [27]	63	Protein (IHC)	57.1%	1.60 (0.65–3.93)
Loos et al. [28]	40	mRNA	50%	4.67 (1.97–11.06)
Geng et al. [29]	40	Protein and mRNA	55%	Not Reported
Birnbaum et al. [30]	453	mRNA	19%	2.22 (1.48–3.33)

**Table 2:** Completed Clinical trials of the PD-1/PD-L1 blockade in PDAC.

Study	Number of Patients	Phase	Study Medication	Cancer Type	Results
Brahmer et al. [66]	207 (14 patients with PDAC)	1	Anti PD-L1	Non-small cell lung cancer, melanoma, renal cell cancer, ovarian cancer, colorectal cancer, pancreatic cancer, gastric cancer and breast cancer	No objective response
Patnaik et al. [67]	30 (one patient with PDAC)	1	Pembrolizumab	Colorectal, melanoma, merkel cell carcinoma, non-small cell lung cancer, prostate cancer, Kaposi sarcoma, soft tissue sarcoma, pancreatic adenocarcinoma	No objective response
Nesselhut et al. [68]	7 (all PDAC)	1	Nivolumab plus dendritic cells	Metastatic pancreatic cancer	2 patients with partial response (PR)
Weiss et al. [69]	17 (11 evaluable chemo naïve patients)	1b/II	Gemcitabine, Nab-Paclitaxel and Pembrolizumab	Metastatic pancreatic cancer	PR for 3 patients and Stable disease for 8 patients

**Table 3:** Selected ongoing trials evaluating anti-PD-1/PD-L1 agents in advanced pancreatic cancer.

Clinical Trials Identifier	Study Phase	Stage of Disease	Study Arm(s)	Endpoint
NCT02648282	2	Locally advanced	Cyclophosphamide (CY) + GVAX + Pembrolizumab + Stereotactic body radiation therapy (SBRT)	Distant metastasis free Survival
NCT03336216	2	Advanced	Investigator choice chemotherapy (Arm A) Nivolumab and CSF1R antibody Cabiralizumab (Arm B) Nivolumab, cabiralizumab plus Gemcitabine/nab-paclitaxel (Arm C) Nivolumab, cabiralizumab plus FOLFOX	mPFS
NCT03190265	2	Advanced	CY+Nivolumab+Ipilumumab + GVAX vaccine + CRS-207 (Arm A) Nivolumab + Ipilumumab + CRS-207 (Arm B)	ORR
NCT02451982	1/2	Resectable PDAC	CY/GVAX (Arm A) CY/GVAX with Nivolumab (Arm B)	Median IL17A expression in vaccine-induced lymphoid aggregates. SE: OS and DFS
NCT02558894	2	Advanced	Anti PD-L1 agent Durvalumab (Arm A) Durvalumab plus anti CTLA-4 Tremelimumab (Arm B)	ORR SE: PFS, OS, best objective response, disease control rate
NCT02309177	1	Advanced	Nab-Paclitaxel and Nivolumab (Arm A) Nab-Paclitaxel, Gemcitabine, and Nivolumab (Arm B)	Safety and dose-limiting toxicity
NCT02303990	1	Advanced (other cancers included)	Pembrolizumab plus hypofractionated radiation therapy	Number of adverse events
NCT02546531	1	Advanced (other cancers included)	Pembrolizumab + Gemcitabine and Defactinib (FAK inhibitor)	Safety and toxicity, ORR, PFS
NCT01714739	1/2	Advanced Solid Tumors	A Study of an Anti-KIR Antibody Lirilumab in Combination with an Anti-PD1 Antibody Nivolumab and Nivolumab Plus an Anti-CTLA-4 Ipilimumab Antibody in Patients with Advanced Solid Tumors	Safety and tolerability and ORR
NCT02311361	1/2	Unresectable Pancreatic Cancer	Immune Checkpoint Inhibition (Tremelimumab and/or MEDI4736) in Combination with Radiation Therapy in Patients with Unresectable Pancreatic Cancer	Safety and ORR
NCT03404960	1/2	Advanced Pancreatic Cancer	Niraparib + Ipilimumab or Nivolumab in Progression Free Pancreatic Adenocarcinoma After Platinum-Based Chemotherapy (Parpvax)	PFS
NCT03006302	2	Metastatic pancreatic Cancer	Epacadostat, Pembrolizumab, and CRS-207, With or Without CY/GVAX Pancreas in Patients with Metastatic Pancreas Cancer	Dose, survival

mPFS: median progression-free survival; ORR: objective response rate; OS: overall survival; DFS: disease free survival; SE: secondary endpoints.

## References

[1] Siegel R.L., Miller K.D., Jemal A. (2018). Cancer statistics, 2018. CA Cancer J. Clin..

[2] Surveillance, Epidemiology, and End results (SEER) Cancer Stat Fcts: Pancreatic Cancer. https://seer.cancer.gov/statfacts/html/pancreas.html.

[3] Liu Q., Liao Q., Zhao Y. (2017). Chemotherapy and tumor microenvironment of pancreatic cancer. Cancer Cell Int..

[4] Zheng L., Xue J., Jaffee E.M., Habtezion A. (2013). Role of immune cells and immune-based therapies in pancreatitis and pancreatic ductal adenocarcinoma. Gastroenterology.

[5] Moertel C.G. (1978). Chemotherapy of gastrointestinal cancer. N. Engl. J. Med..

[6] Burris H.A., Moore M.J., Andersen J., Green M.R., Rothenberg M.L., Modiano M.R., Cripps M.C., Portenoy R.K., Storniolo A.M., Tarassoff P. (1997). Improvements in survival and clinical benefit with gemcitabine as first-line therapy for patients with advanced pancreas cancer: A randomized trial. J. Clin. Oncol..

[7] Conroy T., Desseigne F., Ychou M., Bouche O., Guimbaud R., Becouarn Y., Adenis A., Raoul J.L., Gourgou-Bourgade S., de la Fouchardiere C. (2011). FOLFIRINOX versus gemcitabine for metastatic pancreatic cancer. N. Engl. J. Med..

[8] Von Hoff D.D., Ervin T., Arena F.P., Chiorean E.G., Infante J., Moore M., Seay T., Tjulandin S.A., Ma W.W., Saleh M.N. (2013). Increased survival in pancreatic cancer with nab-paclitaxel plus gemcitabine. N. Engl. J. Med..

[9] Swann J.B., Smyth M.J. (2007). Immune surveillance of tumors. J. Clin. Invest..

[10] Dunn G.P., Old L.J., Schreiber R.D. (2004). The three Es of cancer immunoediting. Annu. Rev. Immunol..

[11] Ribas A., Wolchok J.D. (2018). Cancer immunotherapy using checkpoint blockade. Science.

[12] McDermott D.F., Atkins M.B. (2013). PD-1 as a potential target in cancer therapy. Cancer Med..

[13] Katsuya Y., Fujita Y., Horinouchi H., Ohe Y., Watanabe S., Tsuta K. (2015). Immunohistochemical status of PD-L1 in thymoma and thymic carcinoma. Lung Cancer.

[14] Nakanishi J., Wada Y., Matsumoto K., Azuma M., Kikuchi K., Ueda S. (2007). Overexpression of B7-H1 (PD-L1) significantly associates with tumor grade and postoperative prognosis in human urothelial cancers. Cancer Immunol. Immunother..

[15] Nomi T., Sho M., Akahori T., Hamada K., Kubo A., Kanehiro H., Nakamura S., Enomoto K., Yagita H., Azuma M. (2007). Clinical significance and therapeutic potential of the programmed death-1 ligand/programmed death-1 pathway in human pancreatic cancer. Clin Cancer Res..

[16] Fay A.P., Signoretti S., Callea M., Telomicron G.H., McKay R.R., Song J., Carvo I., Lampron M.E., Kaymakcalan M.D., Poli-de-Figueiredo C.E. (2015). Programmed death ligand-1 expression in adrenocortical carcinoma: An exploratory biomarker study. J. Immunother. Cancer.

[17] Strome S.E., Dong H., Tamura H., Voss S.G., Flies D.B., Tamada K., Salomao D., Cheville J., Hirano F., Lin W. (2003). B7-H1 blockade augments adoptive T-cell immunotherapy for squamous cell carcinoma. Cancer Res..

[18] Jacobs J.F., Idema A.J., Bol K.F., Nierkens S., Grauer O.M., Wesseling P., Grotenhuis J.A., Hoogerbrugge P.M., de Vries I.J., Adema G.J. (2009). Regulatory T cells and the PD-L1/PD-1 pathway mediate immune suppression in malignant human brain tumors. Neuro. Oncol..

[19] Wilmotte R., Burkhardt K., Kindler V., Belkouch M.C., Dussex G., Tribolet N., Walker P.R., Dietrich P.Y. (2005). B7-homolog 1 expression by human glioma: A new mechanism of immune evasion. Neuroreport.

[20] Dong H., Strome S.E., Salomao D.R., Tamura H., Hirano F., Flies D.B., Roche P.C., Lu J., Zhu G., Tamada K. (2002). Tumor-associated B7-H1 promotes T-cell apoptosis: A potential mechanism of immune evasion. Nat. Med..

[21] Jiang Y., Li Y., Zhu B. (2015). T-cell exhaustion in the tumor microenvironment. Cell Death Dis..

[22] Wang X., Teng F., Kong L., Yu J. (2016). PD-L1 expression in human cancers and its association with clinical outcomes. Onco. Targets Ther..

[23] Gao H.L., Liu L., Qi Z.H., Xu H.X., Wang W.Q., Wu C.T., Zhang S.R., Xu J.Z., Ni Q.X., Yu X.J. (2018). The clinicopathological and prognostic significance of PD-L1 expression in pancreatic cancer: A meta-analysis. Hepatobiliary Pancreat. Dis. Int..

[24] Coppock J.D., Volaric A.K., Mills A.M., Gru A.A. (2018). Concordance levels of PD-L1 expression by immunohistochemistry, mRNA in situ hybridization, and outcome in lung carcinomas. Hum. Pathol..

[25] Gerlinger M., Rowan A.J., Horswell S., Math M., Larkin J., Endesfelder D., Gronroos E., Martinez P., Matthews N., Stewart A. (2012). Intratumor heterogeneity and branched evolution revealed by multiregion sequencing. N. Engl. J. Med..

[26] Wang L., Ma Q., Chen X., Guo K., Li J., Zhang M. (2010). Clinical significance of B7-H1 and B7-1 expressions in pancreatic carcinoma. World J. Surg..

[27] Chen Y., Sun J., Zhao H., Zhu D., Zhi Q., Song S., Zhang L., He S., Kuang Y., Zhang Z. (2014). The coexpression and clinical significance of costimulatory molecules B7-H1, B7-H3, and B7-H4 in human pancreatic cancer. Onco. Targets Ther..

[28] Loos M., Giese N.A., Kleeff J., Giese T., Gaida M.M., Bergmann F., Laschinger M., Büchler M.W., Friess H. (2008). Clinical significance and regulation of the costimulatory molecule B7-H1 in pancreatic cancer. Cancer Lett..

[29] Geng L., Huang D., Liu J., Qian Y., Deng J., Li D., Hu Z., Zhang J., Jiang G., Zheng S. (2008). B7-H1 up-regulated expression in human pancreatic carcinoma tissue associates with tumor progression. J. Cancer Res. Clin. Oncol..

[30] Birnbaum D.J., Finetti P., Lopresti A., Gilabert M., Poizat F., Turrini O., Raoul J.L., Delpero J.R., Moutardier V., Birnbaum D. (2016). Prognostic value of PDL1 expression in pancreatic cancer. Oncotarget.

[31] Zhang C.M., Lv J.F., Gong L., Yu L.Y., Chen X.P., Zhou H.H., Fan L. (2016). Role of Deficient Mismatch Repair in the Personalized Management of Colorectal Cancer. Int. J. Environ. Res. Public Health.

[32] Le D.T., Durham J.N., Smith K.N., Wang H., Bartlett B.R., Aulakh L.K., Lu S., Kemberling H., Wilt C., Luber B.S. (2017). Mismatch repair deficiency predicts response of solid tumors to PD-1 blockade. Science.

[33] Kawakami H., Zaanan A., Sinicrope F.A. (2015). Microsatellite instability testing and its role in the management of colorectal cancer. Curr. Treat. Options Oncol..

[34] Boland C.R., Thibodeau S.N., Hamilton S.R., Sidransky D., Eshleman J.R., Burt R.W., Meltzer S.J., Rodriguez-Bigas M.A., Fodde R., Ranzani G.N. (1998). A National Cancer Institute Workshop on Microsatellite Instability for cancer detection and familial predisposition: Development of international criteria for the determination of microsatellite instability in colorectal cancer. Cancer Res..

[35] Salem M.E., Puccini A., Grothey A., Raghavan D., Goldberg R.M., Xiu J., Korn W.M., Weinberg B.A., Hwang J.J., Shields A.F. (2018). Landscape of Tumor Mutation Load, Mismatch Repair Deficiency, and PD-L1 Expression in a Large Patient Cohort of Gastrointestinal Cancers. Mol. Cancer Res..

[36] Champiat S., Ferte C., Lebel-Binay S., Eggermont A., Soria J.C. (2014). Exomics and immunogenics: Bridging mutational load and immune checkpoints efficacy. Oncoimmunology.

[37] NCCN Flash updae: Pancreatic Adenocarcinoma. https://www.nccn.org/about/news/ebulletin/ebulletindetail.aspx?ebulletinid=1193.

[38] Lindor N.M., Burgart L.J., Leontovich O., Goldberg R.M., Cunningham J.M., Sargent D.J., Walsh-Vockley C., Petersen G.M., Walsh M.D., Leggett B.A. (2002). Immunohistochemistry versus microsatellite instability testing in phenotyping colorectal tumors. J. Clin. Oncol..

[39] Hu Z.I., Shia J., Stadler Z.K., Varghese A.M., Capanu M., Salo-Mullen E., Lowery M.A., Diaz L.A., Mandelker D., Yu K.H. (2018). Evaluating Mismatch Repair Deficiency in Pancreatic Adenocarcinoma: Challenges and Recommendations. Clin. Cancer Res..

[40] Kim S.T., Klempner S.J., Park S.H., Park J.O., Park Y.S., Lim H.Y., Kang W.K., Kim K.M., Lee J. (2017). Correlating programmed death ligand 1 (PD-L1) expression, mismatch repair deficiency, and outcomes across tumor types: Implications for immunotherapy. Oncotarget.

[41] Eatrides J.M., Coppola D., Diffalha S.A., Kim R.D., Springett G.M., Mahipal A. (2016). Microsatellite instability in pancreatic cancer. J. Clin. Oncol..

[42] Gryfe R., Kim H., Hsieh E.T., Aronson M.D., Holowaty E.J., Bull S.B., Redston M., Gallinger S. (2000). Tumor microsatellite instability and clinical outcome in young patients with colorectal cancer. N. Engl. J. Med..

[43] Dos Santos N.R., Seruca R., Constancia M., Seixas M., Sobrinho-Simoes M. (1996). Microsatellite instability at multiple loci in gastric carcinoma: Clinicopathologic implications and prognosis. Gastroenterology.

[44] Achille A., Biasi M.O., Zamboni G., Bogina G., Iacono C., Talamini G., Capella G., Scarpa A. (1997). Cancers of the papilla of vater: Mutator phenotype is associated with good prognosis. Clin. Cancer Res..

[45] Paulson T.G., Wright F.A., Parker B.A., Russack V., Wahl G.M. (1996). Microsatellite instability correlates with reduced survival and poor disease prognosis in breast cancer. Cancer Res..

[46] Zhou X., Kemp B.L., Khuri F.R., Liu D., Lee J.J., Wu W., Hong W.K., Mao L. (2000). Prognostic implication of microsatellite alteration profiles in early-stage non-small cell lung cancer. Clin. Cancer Res..

[47] Nakata B., Wang Y.Q., Yashiro M., Nishioka N., Tanaka H., Ohira M., Ishikawa T., Nishino H., Hirakawa K. (2002). Prognostic value of microsatellite instability in resectable pancreatic cancer. Clin. Cancer Res..

[48] Yamamoto H., Itoh F., Nakamura H., Fukushima H., Sasaki S., Perucho M., Imai K. (2001). Genetic and clinical features of human pancreatic ductal adenocarcinomas with widespread microsatellite instability. Cancer Res..

[49] Lee L.H., Cavalcanti M.S., Segal N.H., Hechtman J.F., Weiser M.R., Smith J.J., Garcia-Aguilar J., Sadot E., Ntiamoah P., Markowitz A.J. (2016). Patterns and prognostic relevance of PD-1 and PD-L1 expression in colorectal carcinoma. Mod. Pathol..

[50] Lemery S., Keegan P., Pazdur R. (2017). First FDA Approval Agnostic of Cancer Site - When a Biomarker Defines the Indication. N. Engl. J. Med..

[51] Le D.T., Kavan P., Kim T.W., Burge M.E., Van Cutsem E., Hara H., Boland P.M., Van Laethem J.-L., Geva R., Taniguchi H. (2018). KEYNOTE-164: Pembrolizumab for patients with advanced microsatellite instability high (MSI-H.) colorectal cancer. J. Clin. Oncol..

[52] El-Khoueiry A.B., Sangro B., Yau T., Crocenzi T.S., Kudo M., Hsu C., Kim T.Y., Choo S.P., Trojan J., Welling T.H.R. (2017). Nivolumab in patients with advanced hepatocellular carcinoma (CheckMate 040): An open-label, non-comparative, phase 1/2 dose escalation and expansion trial. Lancet.

[53] Evans R.A., Diamond M.S., Rech A.J., Chao T., Richardson M.W., Lin J.H., Bajor D.L., Byrne K.T., Stanger B.Z., Riley J.L. (2016). Lack of immunoediting in murine pancreatic cancer reversed with neoantigen. JCI Insight.

[54] Alexandrov L.B., Nik-Zainal S., Wedge D.C., Aparicio S.A., Behjati S., Biankin A.V., Bignell G.R., Bolli N., Borg A., Borresen-Dale A.L. (2013). Signatures of mutational processes in human cancer. Nature.

[55] Vonderheide R.H., Bayne L.J. (2013). Inflammatory networks and immune surveillance of pancreatic carcinoma. Curr. Opin. Immunol..

[56] von Bernstorff W., Spanjaard R.A., Chan A.K., Lockhart D.C., Sadanaga N., Wood I., Peiper M., Goedegebuure P.S., Eberlein T.J. (1999). Pancreatic cancer cells can evade immune surveillance via nonfunctional Fas (APO-1/CD95) receptors and aberrant expression of functional Fas ligand. Surgery.

[57] Nowak A.K., Robinson B.W., Lake R.A. (2003). Synergy between chemotherapy and immunotherapy in the treatment of established murine solid tumors. Cancer Res..

[58] Plate J.M., Plate A.E., Shott S., Bograd S., Harris J.E. (2005). Effect of gemcitabine on immune cells in subjects with adenocarcinoma of the pancreas. Cancer Immunol. Immunother..

[59] Lutz E.R., Wu A.A., Bigelow E., Sharma R., Mo G., Soares K., Solt S., Dorman A., Wamwea A., Yager A. (2014). Immunotherapy converts nonimmunogenic pancreatic tumors into immunogenic foci of immune regulation. Cancer Immunol. Res..

[60] Le D.T., Ko A.H., Wainberg Z.A., Picozzi V.J., Kindler H.L., Wang-Gillam A., Oberstein P.E., Morse M., Zeh H., Weekes C.D. (2017). Results from a phase 2b, randomized, multicenter study of GVAX pancreas and CRS-207 compared to chemotherapy in adults with previously-treated metastatic pancreatic adenocarcinoma (ECLIPSE Study). J. Clin. Oncol..

[61] Ribas A., Dummer R., Puzanov I., VanderWalde A., Andtbacka R.H.I., Michielin O., Olszanski A.J., Malvehy J., Cebon J., Fernandez E. (2017). Oncolytic Virotherapy Promotes Intratumoral T Cell Infiltration and Improves Anti-PD-1 Immunotherapy. Cell.

[62] Puzanov I., Milhem M.M., Minor D., Hamid O., Li A., Chen L., Chastain M., Gorski K.S., Anderson A., Chou J. (2016). Talimogene Laherparepvec in Combination with Ipilimumab in Previously Untreated, Unresectable Stage IIIB-IV Melanoma. J. Clin. Oncol..

[63] Inman K.S., Francis A.A., Murray N.R. (2014). Complex role for the immune system in initiation and progression of pancreatic cancer. World J. Gastroenterol..

[64] Koblish H.K., Hansbury M., Wang L.-C.S., Yang G., Huang T., Xue C.-B., Li Y.-L., Yue E., Combs A., Yao W. (2015). Abstract 1336: Novel immunotherapeutic activity of JAK and PI3Kδ inhibitors in a model of pancreatic cancer. Cancer Research.

[65] Hingorani S.R., Harris W.P., Hendifar A.E., Bullock A.J., Wu X.W., Huang Y., Jiang P. (2015). High response rate and PFS with PEGPH20 added to nab-paclitaxel/gemcitabine in stage IV previously untreated pancreatic cancer patients with high-HA tumors: Interim results of a randomized phase II study. J. Clin. Oncol..

[66] Brahmer J.R., Tykodi S.S., Chow L.Q., Hwu W.J., Topalian S.L., Hwu P., Drake C.G., Camacho L.H., Kauh J., Odunsi K. (2012). Safety and activity of anti-PD-L1 antibody in patients with advanced cancer. N. Engl. J. Med..

[67] Patnaik A., Kang S.P., Rasco D., Papadopoulos K.P., Elassaiss-Schaap J., Beeram M., Drengler R., Chen C., Smith L., Espino G. (2015). Phase I Study of Pembrolizumab (MK-3475; Anti-PD-1 Monoclonal Antibody) in Patients with Advanced Solid Tumors. Clin. Cancer Res..

[68] Nesselhut J., Marx D., Lange H., Regalo G., Cillien N., Chang R.Y., Nesselhut T. (2016). Systemic treatment with anti-PD-1 antibody nivolumab in combination with vaccine therapy in advanced pancreatic cancer. J. Clin. Oncol..

[69] Weiss G.J., Blaydorn L., Beck J., Bornemann-Kolatzki K., Urnovitz H., Schutz E., Khemka V. (2018). Phase Ib/II study of gemcitabine, nab-paclitaxel, and pembrolizumab in metastatic pancreatic adenocarcinoma. Invest. New Drugs.

